# Seroprevalence of *Toxoplasma gondii* among pregnant women attending antenatal clinics at Hawassa University comprehensive specialized and Yirgalem General Hospitals, in Southern Ethiopia

**DOI:** 10.1186/s12879-019-4694-8

**Published:** 2019-12-16

**Authors:** Demissie Assegu Fenta

**Affiliations:** College of Medicine and Health Sciences, School of Medical Laboratory Science, Hawassa University, P.O. Box.1560, Hawassa, Ethiopia

**Keywords:** T. Gondii, Pregnant women, Hawassa, Yirgalem, Ethiopia

## Abstract

**Background:**

Toxoplasmosis is caused by infection with the protozoan parasite *Toxoplasma gondii*. It is acquired by consumption of raw or undercooked meat containing tissue cyst, food or water contaminated with oocyst and congenital infection through the placenta leading to serious congenital abnormalities in the fetus like miscarriage, stillbirth, intrauterine death and neurologic defects. Therefore; this study was aimed to determine the prevalence of toxoplasmosis infection and its possible risk factors associated with pregnant women attending antenatal clinics in Hawassa and Yiregalem Hospitals, Southern Ethiopia.

**Methods:**

A hospital-based cross-sectional study was conducted from December 2016 to May 2017. The study was done in antenatal care clinics of Hawassa and Yiregalem Hospitals in Southern, Ethiopia. Five hundred pregnant women were interviewed with a pretested structured questionnaire to collect risk factors and socio-demographic data. Blood samples were collected and serum was separated and tested for a*nti- Toxoplasma gondii* antibodies using ELISA (Enzyme-linked Immunosorbent Assay). Data were analyzed using SPSS version 20 statistical software. The risk factors were tested for significance using Bivariate and multivariate analysis. *P*-value < 0.05 was considered statistically significant.

**Results:**

The weighted prevalence of this study was 81.8% for the anti- *Toxoplasma gondii* antibody. Almost all participants (99.6%) had no information about the disease. A significant association was observed between seroprevalence and contact with domestic cats (OR = 1.206, 95% CI (1.627–2.206, *P* = 0.043), consumption of raw meat (OR = 0.848, 95% CI: 1.517–2.941, *P* = 0.019) and unpasteurized milk (OR = 0.871, 95% CI 1.531–2.221, *P* = 0.032). A significant association was not observed between seroprevalence and age, history of abortion, and blood transfusion.

**Conclusions:**

The findings of this study demonstrated a relatively higher prevalence of seropositivity than studies reported from other countries. Existence of domestic cats at home, consumption of undercooked meat and unpasteurized milk were identified as risk factors for *T. gondii* infection. Therefore, a health education program to increase the mother’s knowledge about *toxoplasmosis* towards avoiding eating undercooked meat, contact with cats and consumption of unpasteurized milk during pregnancy is recommended. Furthermore, our results suggested that the implementation of newborn screening and follow-up testing can lead to reducing of toxoplasmosis associated complications.

## Background

*Toxoplasma gondii (T. gondii)* is an obligate single-celled, intracellular protozoan parasite belonging to phylum Apicomplexa that causes a zoonotic infectious disease, toxoplasmosis which can infect wide-ranging warm-blooded vertebrates such as human as well as other warm-blooded domestic and wild animals [1–3]. This parasitic infection is a neglected disease out of five parasitic infections which have been classified as a concern to public health by Center for disease control (CDC) or a member of TORCH group infectious agents; consisting of Rubella, Cytomegalovirus, Herpes viruses and *Treponema pallidum*, which causes infection of the fetus transplacentally with congenital abnormalities, and even fetal loss both in humans and animals [4].

The infection has a worldwide prevalence of about one-third of the population all over the world to be exposed to this parasite. However; the incidence rate of 400–4000 congenital toxoplasmosis cases per year has been reported [5] Its prevalence have been reported from various parts of the world such as United State 15% from childbearing age women, France (44%) and Indonesia greater than 60% from pregnant women, 30–75% in Latin America, parts of Eastern/Central Europe, the Middle East, parts of south-east Asia and Africa from pregnant women [6].

The prevalence has been declining in parts of Eastern and central Europe over the past three to four decades [7] High prevalence of the infection have been reported from pregnant women and women of childbearing age in Latin America, the Middle East, parts of south-east Asia [6] In Africa, particularly in Sub-Saharan Africa the prevalence of *T. gondii* is increased in association with HIV [8] . Different prevalence values were reported from different African countries such as Ghana 92.5% [9], and Sudan 79.8% [10]. Most pregnant women infected with *T. gondii* are chronically infected while few acquire the infection. However; pregnant women with acute infection during pregnancy are at risk of congenitally transmitting the infection to the fetus [11].

In Ethiopia, the seroprevalence *T.gondii* range from 20.2% from the general population to 90% among HIV patients [8] According to the Ethiopian Demographic and Health Survey (EDHS 2016) the infant mortality rate was 48 deaths per 1000 live birth among these deaths 28 of them were due to infection [12] Furthermore; studies from large hospitals in the country also reported higher prevalence rates of *T.gondii* 83.6% from Jimma [13] 88.6% Gondar [14], 85.4% Addis Abeba [15], 81.4% Central Ethiopia [16], 79.3% Arba Minch hospital [17] and 85.3% from Bench Maji zone [18].

This parasite has a wide host range, infecting most warm-blooded species but the life cycle is completed only in felis [4] humans usually become infected by ingesting of raw or undercooked meat containing tissue cysts, drinking of unpasteurized milk, ingestion of vegetables contaminated with soil, water and food with sporulated oocysts shed-cat faece [9].

This parasite is primarily very significant in pregnancy as it can cross the placental barrier to infect the fetal tissues and thereby cause congenital deformities such as hydrocephaly, mental retardation or chorioretinitis [19–21]. There are rare occasions of transmission through tachyzoites contained in blood products during (blood transfusion, organ or tissue transplantation), consumption of vegetables and needle-stick injuries or cuts [4]. Risk group individuals like laboratory workers who handle infected blood can also acquire infection through accidental inoculation [4]. In Immunocompetent individuals, *T. gondii* causes a latent infection characterized by the persistence of the organism primarily in the brain, skeletal muscle, and heart tissues without causing clinical symptoms. However, in chronically infected individuals with impaired cell-mediated immunity symptomatic disease more likely occurs as a result of the recurrence of latent infection [22, 23].

Individuals with a weakened immune system, such as those infected with HIV and Pregnant women may become seriously ill, and infection can occasionally be fatal and also results in hospitalization [4, 12] If the diagnosis is delayed, it might have a great contribution as it has the ability to cause, spontaneous abortion, stillbirth and preterm deliveries [15] and unavoidable and irreversible fetal damage might also take place [24, 25].

In spite of the widespread practice of keeping cats as domestic animals and the presence of stray cats, the habit of eating raw meat, consumption of unpasteurized milk and suitable climatic conditions favors the survival of the parasite in the study area [26, 27]. To the best of our knowledge, there is no regular serological screening of pregnant women for *T. gondii* infection. Moreover, there is no documented data about the prevalence of the disease and associated risk factors in the study area. It is believed that antenatal data on this parasitic disease in the study area would give baseline information about the prevalence of *T. gondii* in pregnant women and also for the planning and implementation of *T. gondii* comprehensive diagnostic, control and prevention strategies. Therefore, this study was aimed to determine the seroprevalence of *T. gondii* infection and its risk factors among pregnant women attending ANC clinics’ on the emphasis of screening and management of pregnant women in Hawassa University and Yirgalem hospitals, Southern Ethiopia.

## Methods

### Study design, area and period

The cross-sectional hospital-based study was conducted from December 2016–March 2017 among pregnant women to assess the seroprevalence of *T. gondii* at Yirgalem and Hawassa University comprehensive Specialized Hospitals ANC clinics. These two hospitals are located in Southern Nations and Nationalities People’s Region (SNNPR), 345 and 275kms apart from Addis Abeba the capital city of Ethiopia respectively. While Yirgalem hospital is 70kms from the regional capital city Hawassa. These two larger hospitals of the region are serving as teaching, training and clinical service centers. The ANC clinic of Yirgalem Hospital serves more than 25 pregnant women per day with 69 beds for both prenatal and postnatal services. However, 45 pregnant women were visiting Hawassa University Hospital ANC clinic per day and about 200 beds are giving service for both prenatal and postnatal services.

### Study population

A total of 500 pregnant women attending ANC clinics of Yirgalem and Hawassa University comprehensive Specialized Hospitals were our study population. Pregnant women who had emergency conditions and requiring urgent intervention and who refused to give consent were not included in the study.

### Sample size and sampling technique

Sample size was determined using single population proportion formula as 500 based on a prevalence of 43.9% taken from a study conducted in Bench Maji zone, Ethiopia [17], with 95% confidence interval and a maximum discrepancy of 10% between the sample and the underline population for Yirgalem hospital gives 193 pregnant women and prevalence 81% from a study conducted in Jimma to select 307 pregnant women from Hawassa university hospital [13]. Finally, a total of 500 pregnant women were interviewed. Of these 6 of them were no responses.

### Data collection

Socio-demographic characteristics and data on exposure to possible risk factors were collected for each study participant using a structured questionnaire consisting of consumption of raw meat and unwashed raw vegetables, presence of a domestic cat at home, history of abortion, blood transfusions and others.

Venous blood samples (5 ml) were collected for a routine antenatal check-up and for our study after obtaining consent from each study participants by strictly following the standard operating procedure in place. The blood samples were clotted for 30 min at room temperature and centrifuged at 3000 rpm for 5 min to separate serum which was kept at − 20 °C in the refrigerator until it was transported to the laboratory and serological tests were carried out. The questionnaire was pretested at Adare General Hospital before data collection and administered orally before blood sample collection and checked for its consistency.

The serum was tested for *anti-T. gondii* IgM and IgG antibodies using Enzyme-Linked Immunosorbent Assay following the method of Engvall and his colleagues with the commercial ENZYWELL TOXOPLASMA IgG and IgM KIT, using (Siemens Healthcare Diagnostics Products GmbH, Marburg, Germany). According to the manufacturer’s instructions (Engvall and Perlmann, 1971, 1972). The different values of the calibrator identified the upper limit of the reference range of non-infected individuals (Cut-off). In addition to that, the findings were evaluated quantitatively by calculating a ratio of the extinction value of the control or patient samples over the extinction value of the calibrator. If the absorbance of the sample is higher than that of the Cut-off (> 1.3 for IgG and > 1.2 for IgM), the sample was considered positive for the presence of specific antibodies and negative if it was < 0.7 of IgG and < 0.8 for IgM. In case borderline test results of both (0.7–1.3 for IgG and 0.8–1.2 for IgM), tests were repeated.

### Statistical analysis

All collected data from the questionnaire and laboratory were checked for completeness and consistency, entered into a computer, cleaned and analyzed using SPSS version 20 software package. Descriptive statistics were used to calculate the demographic data of the study participants. Bivariate and multivariate logistic regressions were used to assess the association between potential risk factors considered and *T. gondii* infection. Variables with *P*-value < 0.25 by the bivariate analysis were entered into the multivariate model. A multivariate logistic regression, *p*-value < 0.05 was considered as statistically significant for all variables.

### Ethical consideration

The study was ethically cleared by the Institutional Review Board (IRB) of Hawassa University College of medicine and health science on 14 February 2017 and approved the proposal with (Reference number: IRB -057-14- 02/2017). An original copy of the approval, as well as the consent form, is available upon request.. Permission letter was obtained from SNNPR Sidama Zone Health Bureau, Yirgalem and Hawassa University hospital administrations.

The purpose and importance of the study were explained to each study participant and parents and guardians of a few minorities. Finally, consent instead of assent was secured from all participants including minor participants. Permission was obtained from parents or guardians to obtained consent from minor participants who have ethical dilemmas based on the Ethiopian national research Ethics review guideline. Furthermore, the minor participant should be given appropriate information based on the participant’s level of comprehension and where early marriage is common like our study area. Therefore; consent was taken from each participant who was under the age of 18 years with a participant affirmative agreement to participate in the research according to the guideline and for the purpose of data quality. To ensure confidentiality of participant’s information, anonymous typing was applied.

## Result

### Socio-demographic characteristics

A total of 494 pregnant women age between 15 to 42 years with a mean age of 4.6 ± 26.47 years attending ANC clinics of Yirgalem and Hawassa University hospital were selected and participated in the study. The majority (60.8%) of the study participants were found within the age group of 25–34.9 years. Almost all, (99.6%) of the participants had no information about the disease *toxoplasmosis* and its mode of transmission. Of all participants, 333 (67.5%) and 161(32.5%) were urban and rural residents respectively. According to their gestational age 101(20.4%), 180(36.4%) and 213(43.2%) were at first^,^ second and third trimester respectively. 168(53.9%) of participants had at least elementary education. 446(90.3%) were married and 246(49.7%) were housewives. The seroprevalence of *T. gondii* and other demographic characteristics of the pregnant women is indicated in (Table 1).

**Table 1:** Distribution of *T. gondii* infection along with demographic characteristics of the pregnant women (*n* = 494), Hawassa Ethiopia, 2017

Demographic Characteristics	Total n (%)	Seroprevalence		*P*-Value	COR 95% (CI)	*P*-Value	AOR 95% (CI)
		Negative n (%)	Positive n (%)				
Age group (in Years)							
15–24.9	161(%)	34(37.8%)	127(31.6%)	0.501	0.49(0.486–1.505)	0.482	0.69(0.248–1.951)
25–34.9	301(60.8%)	51(56.7%)	250(61.9%)		0.51(0.499–1.519)	0.860	0.85(0.334–2.459)
> 35^a^	32(6.5%)	5(5.6%)	27(6.7%)		0.53(0.524–1.543)		
Residence							
Urban	333(67.5)	57(63.3%)	276(68.3%)	0.001	0.93(1.21–2.30)	0.001	1.25(1.043–2.012)
Rural^a^	161(32.5%)	33(36.7%)	128(31.7%)		1.16(0.851–1.573)		
Education status							
Illiterate	59(11.9%)	8(8.9%)	51(12.6%)	0.595	0.69(0.589–1.608)	0.697	1.20(0.480–3.002)
Elementary	168(53.9%)	32(35.6%)	136(33.7%)		0.59(0.578–2.597)	0.507	0.80(0.414–1.545)
Secondary	166(33.5)	34(37.8%)	132(32.7%)		0.63(0.619–1.638)	0.347	0.73(0.380–1.405)
College and above^a^	101(20.4)	16(17.8%)	85(21%)		0.85(0.846–2.860)		
Marital status							
Married	446(90.3%)	81(90%)	365(90.3%)	0.697	0.73(0.698–2.716)	0.004	-
Single	30(6.1%)	5(5.6%)	25(6.2%)		0.88(0.672–2.690)	1.00	-
Divorced	15(3%)	4(4.4%)	11(2.7%)		0.89(0.782–1.798)	1.00	–
Widowed^a^	3(0.6%)	0(0%)	3(0.7%)		1.00		
Occupation							
Housewife	246(49.7%)	44(48.9%)	202(50%)	0.542	0.64(0.531–1.551)	0.584	1.29(0.523–3.160)
Health worker	19(3.8%)	3(3.3%)	16(4%)		1.00	0.598	1.49(0.336–6.633)
Employed	133(26..9%)	28(31.1%)	105(26%)		0.52(0.508–1.528)	0.919	1.05(0.412–2.677)
Daily labor	12(2.4%)	3(3.3%)	9(2.2%)		1.00	0.826	0.84(0.178–3.967)
Merchant	52(10.5%)	5(5.6%)	47(11.6%)		0.58(0.469–2.489)	0.128	0.84(0.178–3.967)
Student^a^	32(6.5%)	7(7.8%)	25(6.2%)		0.86(0.851–1.865)		
Gestational age							
1st trimester	101(20.4%)	22(24.4%)	79(19.6%)	0.019	1.04(1.723–2.273)	0.001	1.34(1.272-
2nd trimester	180(36.4)	40(44.4%)	140(34.7%)		1.24(1.034–2.041)	0.028	1.532))
3rd trimester^a^	213(43.2%)	28(31.1%)	185(45.8%)		0.41(1.032–1.039)	0.030	1.53(1.312–2.900)
Place of previous birth							
No birth	150(30.3%)	32(35.6%)	118(29.2%)	0.135	0.47(0.459–1.478)	0.315	0.77(0.462–1.282)
Home	95(19.2%)	15(16.7%)	80(19.8%)	0.200	0.48(0.459–1.478)	0.013	1.59(1.113–2.115)
Health institution^a^	249(50.5%)	43(47.8%)	206(51%)		0.49(0.476–1.495)	0.473	

*COR* Crude Odds Ratio, *AOR* Adjusted Odds Ratio, *CI* Confidence Interval, ^a^adjusted variable

### Seroprevalence of *T. gondii* and associated factors

Among 494 participants tested for *anti-T. gondii* IgG and IgM antibodies, 404 were found to be seropositive, giving an overall prevalence of 81.8%. Among the total seropositive 370 (74.9%), 19(3.8%) and 15(3.1%) were positive for Toxoplasma IgG, IgM, and both IgG and IgM respectively. The proportion of IgG−/ IgM-, IgG +/IgM +, IgG−/ IgM+ and IgG +/ IgM- in this study was 124/475,370/19,124/19 and 370/475 respectively. The combination of both immunoglobulins was stratified as IgG and IgM assay expressed as both negative (never infected), IgM only (acute infection), IgM and IgG positive (recent infection) and IgG only (latent infection). The presence of specific IgM is generally suggested to be associated with acute infection. However, this may not be the case in toxoplasmosis, as the continuation of IgM for several months in the sera may interfere in calculating the time of exposure.

Of the total 494 pregnant women, 301(60.8%) were within the age range of 25–34.9 years. Among these 250(61.9%) were positive for anti- *T. gondii* antibody. The prevalence of *T. gondii increases with* age but not significantly associated with *T. gondii* seropositivity (*p* = 0.86). In the current study 59(11.9%) were illiterate among these 51(12.6%) were positive for *the T. gondii* antibody. But there was no significant difference between seropositivity and being illiterate and literate (*P* = 0.595). In this study, a significant difference was not indicated between seropositivity and participants with different occupations (*P* = 0.542). From all participants responded 23(4.7%) have a history of blood transfusion. Of these 18(78.3%) were seropositive for *T. gondii* (Table 1).

Based on their gestational period the distribution of participants was 101(20.4%), 180(36.4%) and 213(43.2%) in the first, second and third trimester respectively. Among these 79(19.6%) 140(34.7%) 185(45.8%) were seropositive for *T. gondii.* Indicated that as the gestational period increases the positivity of *T. gondii*.antibody also showed an increasing trend in this study.

Pregnant women who had close contact with domestic cats were 130(26.3%). Of whom 104(80%) were seropositive for *T. gondii*. Antibody and a significant association between contact with domestic cats at home and *T. gondii* antibody seropositivity were indicated, (OR = 1.206, 95% CI: 1.625–2.206, *P* = 0.043).

In the current study from 291(58.9%) participants who have the habit of eating raw meat 242(83.2%) were positive for *T. gondii* antibodies and there was a significant association between eating raw meat and *T. gondii*. Seropositivity (OR = 0.848, 95% CI 1.517–1.941, *P* = 0.019). Among a total of pregnant women who participated in this study 187(37.8%) have a habit of drinking unpasteurized milk and 153(81.8%) of them were positive for *T. gondii*. Seropositivity and showed a significant association between seropositivity of *T. gondii* and using unpasteurized milk (OR = 0.871, 95% CI, 1.531–2.221, *P* = 0.033). Other risk factors and their significant associations were indicated in the table below (Table 2).

**Table 2:** *Toxoplasmosis* and associated risk factors among pregnant women attending ANC of Yirgalem and Hawassa hospitals southern Ethiopia

Risk factors	*Toxoplasma gondii* Ab.		Total N(%)	*P*-value	COR 95% (CI)	*P*-value	AOR 95% (CI)
	Positive	Negative					
Surgery							
Yes	69(83.1%)	14(16.8%)	83(16.8)	0.727	0.89(0.478–1.673)	0.727	1.12(0.598–2.091)
No^a^	335(81.5%)	76(18.5%)	411(83.2%)				
Blood transfusion							
Yes	18(78.3%)	5 (21.7%)	23(4.7%)	0.654	1.26(0.456–3.492)	0.655	1.26(0.456–3.492)
No^a^	386(82%)	85(18%)	471(95.3%)				
History of abortion							
Yes	52(77.6%)	15(22.4%)	67(13.6%)	0.342	1.35(0.724–2.532)	0.343	1.35(0.724–2.532)
No^a^	352(82.4%)	75(17.6%)	427(86.4%)				
Presence of cats							
Yes	104(80%)	26(20%)	130(26.3%)	0.047	1.21(1.725–2.006)	0.043	1.72(1.625–2.206)
No^a^	301(82.7%)	63(17.3%)	364(73.7%)				
Keep dogs in house							
Yes	124(84.4)	23(15.6%)	147(29.9%)	0.365	0.79(0.468–1.325)	0.366	0.79(0.468–1.323)
No^a^	280(80.9%)	66(19.1%)	346(70%)				
Raw meat eating habit							
Yes	242(83.2%)	49(16.8%)	291(58.9%)	0.036	1.76(1.570–2.209)	0.019	1.65(1.517–1.941)
No^a^	163(80.3%)	40(19.7%)	203(41.1)				
Types of milk							
Unpasteurized	153(81.8%)	34(18.2%)	187(37.8%)	0.015	1.45(1.182–1.688)	0.033	1.87(1.531–2.221)
Pasteurized	196(84.2%)	37(15.8%)	233(47.2%				
Not at all^a^	55(75.3%)	18(24.6%)	74(18.8)				
Raw vegetables							
Yes	334(82.3%)	72(17.7%)	406(82.2%)	0.619	0.60(0.591–1.610)	0.826	0.94(0.535–1.648)
No^a^	71(80.7%)	17(19.3%)	88(17.8%)				
Contact with soil							
Yes	125(79.7%)	32(20.3%)	157(31.8%)	0.475	1.19(0.735–1.936)	0.475	1.19(0.735–1.936)
No^a^	279(82.8%)	58(17.2%)	337(68.2%)				
Sources drinking water							
Pipe water^a^	79(80.6%)	19(19.4%)	98(19.8%)	0.701	1.12(0.636–1.962)	0.701	1.12(0.636–1.962)
	325(82.1%)	71(17.9%)	396(80.2%)				
Slaughter house							
Yes	33(89.2%)	4(10.8%)	37(7.5%)	0.509	0.48(0.472–1.491)	0.342	0.61(0.223–1.681)
No^a^	372(81.4%)	85(18.6%)	457(92.5%)				

*COR* Crude Odds Ratio, *AOR* Adjusted Odds Ratio, *CI* Confidence Interval, ^a^adjusted variable

In the current study, the seropositivity of IgM antibody for pregnant women who have contact with domestic cats and those who have a habit of eating raw meat were significantly associated at (OR = 0.628,95% CI, 1.216–1.560, *P* = 0.001*) and (OR = 0.45, 95% CI, 1.272–1.504, *P* = 0.001) respectively.

## Discussion

This study is one of the few studies carried out in the southern region of Ethiopia to explore the seroprevalence of *T. gondii* infection among pregnant women at Hawassa and Yirgalem hospital.

Toxoplasmosis is a curable but potentially fatal disease and its long term complications following congenital infection and its capacity to cause life-threatening opportunistic infections in immunosuppressed conditions is a major problem in most communities with a high prevalence of *T. gondii* infection [28].

In the present study, there was a non-statistically significant increase in the seropositivity of anti-*T. gondii* antibody in the age group of 25–34.9 (60.8%) (Table 1) study participants. However, the finding was in agreement with other similar studies in which increasing age was more associated with chronic infection [18, 29–31].

In this study 333 (67.5%) and 161(32.5%) were urban and rural residents respectively. However, there were no significant differences in the seroprevalence of anti-*T. gondii* antibodies with the residence. Similar findings were reported from [32–34]. On the other hand, opposite findings were reported from studies conducted in [33–36] between being rural or urban and *T. gondii* seropositivity.

The highest *T.gondii* antibodies were found 213 (43.2%) in those pregnant women in the third trimester and there was a statistically significant association between the gestational age at (first, second and third trimester) and seropositivity of *T.gondii* antibody. This result is in agreement with other studies reported from Ethiopia [17] and Saud Arabia [33] but different reports from this finding were reported from Ethiopia [18, 31], Rwanda [30] and Egypt [37]. In this study 168(53.9%) of the participants had at least elementary education however, no significant difference was observed between the different levels of education. Similar findings were reported from London [7], Ethiopia and Iran [17, 28], Cameroon [36] and Nigeria [38]. Inconsistent findings to this study were reported from studies from Mexico [29] and Ethiopia [39].

Majority 446(90.3%) of participants were married. However, marital status was not significantly associated with *T.gondii* antibody seropositivity. Comparable findings were reported from Ethiopia [18] and Rwanda [30] and the opposite finding was reported from Tobego [40].

The total prevalence of *anti-Toxoplasma gondii* antibodies in pregnant women in the present was 81.8%. The high prevalence of *anti-T. gondii* antibodies observed in current study was in agreement with seroprevalence data from previous studies conducted in Ethiopia: 90% from HIV infected and HIV uninfected individuals [22]; 83.6% among pregnant women in Jimma town [13]; 81.4% among women of childbearing age [16] and other countries 81.2% Saudi Arabia [41], 79.8% Sudan [10] and 74.5% Democratic Republic of Sao Tome [42]. However, our findings, as well as those of previous works in Ethiopia, were substantially higher than other results which were reported from other countries; 34.1% in Sudan [43], 30.9% in Tanzania [34], 40.2% Nigeria [38], 39.3% in Tobago [40], This variation might be attributed to climate and cultural differences and hygienic and feeding habit difference of each country.

The prevalence of the current study was higher than other previous studies reported from different countries Tanzania (30.9%) [34], Nigeria (40.2%) [38], Tobago (39.3%) [40], 14 to 25.7% in Sweden [44], 23.6% in Italy [45], 0.8% in Korean [46] and one study 68.4% in Debre Tabor Ethiopia [47]. On the other hand, higher than the current prevalence were reported from 92.5% in Accra Ghana [48], 88.6% in Gondar Ethiopia [14], 86.4% in Central Ethiopia (86.4%) [16] and 85.3% in Mizan Aman general hospital Ethiopia [18].

This difference might be due to the differences in socioeconomic status, personal hygiene practices, feeding habits, deference in testing methods, variation in the immune status of the study participants and sample size of each study.

In our study, a significant association was observed between seroprevalence and ingestion of raw meat (OR = 0.848, 95% CI 1.517–1.941, *P* = 0.019) and similar findings were reported from other studies in Ethiopia [18, 39], Egypt [37] and Khartoum Sudan [49]. However, inconsistent findings were reported from Ethiopia and Turkey [16, 32] and Mexico [29, 50]. This difference might be due to the difference in socioeconomic status and feeding habits of study participants. Drinking of unpasteurized milk was significantly associated with seropositivity of *T. gondii* (OR = 0.871, 95% CI, 1.531–2.221, *P* = 0.033). Consistent results were reported from London [7], Khyber [33] and Egypt [37]. Inconsistent results in this study were reported from Ethiopia [16, 32], Cameroon [36] and Burkina Faso [43]. This might be due to the difference in the habit of consumption and handling of milk and milk products in the study participants. Although cats are the definitive hosts that shed oocysts, participants who have close contact with domestic cats found to be significantly associated with *T. gondii* infection with *T. gondii* (*P* = 0.044) (OR = 1.206, 95% CI: 0.725–2.006). Comparable findings were reported from Ethiopia [18], Tobago [40] and Brazil [51]. Different findings to this study were reported from Ethiopia [15, 16], Egypt [37], Mexico [50] and Burkina Faso [52].

Frequently eating unwashed and raw fresh fruits and vegetables showed no significant association with *Toxoplasma* infection in this study. (*P* = 0.826) (AOR = 0.939; 95% CI (0.535–1.648).The finding was in agreement with other studies reported from Ethiopia [15, 39], Korean [46] and Mexico [50]. Incomparable results were reported from Buenous Ires [11] Ethiopia [16] and Egypt [37]. This might be due to the difference in feeding habits and personal hygiene practice of the study participants.

Concerning the experience of blood transfusion, no significant difference was observed (*P* = 0.655) (AOR = 1.261(0.456–3.492) among pregnant women who had or did not have a blood transfusion. Furthermore, risk factors like the history of abortion, drinking of unsafe water, contact with dogs working environment like slaughterhouse, surgery, and contact with soil were no significantly associated with *T. gondii* seropositive in this study (Table 2).

### Limitations

We encountered inevitable limitations in the current study in all IgG−/IgM+ participants, follow up serological study was not conducted to prove seroconversion of IgG in these groups of participants. Furthermore; to address the possibility of false-positive IgM results due to the persistence of IgM antibody for several years and *Toxoplasma* gondii IgM diagnostic test errors were not checked. Future follows up studies among pregnant women as well as, newborns will be important for tracing acute congenital Toxoplasmosis and to avoid false-positive reports due to different reasons.

## Conclusion

The findings of this study demonstrated a relatively higher prevalence of seropositivity than studies reported from other countries. Existence of domestic cats at home, consumption of undercooked meat and unpasteurized milk were identified as risk factors for *T. gondii* infection. Therefore, a health education program to increase the mother’s knowledge about *toxoplasmosis* towards the source of infection, modes of transmission and prevention methods by avoiding eating raw or undercooked meat, contact with cats and consumption of unpasteurized milk during pregnancy is recommended. Advocating for the implementation of control and preventive measures and routine screening services for *T. gondii* infection to be integrated with other ANC services to identify potential effects of the infection and its management should be considered as the main strategy to minimize congenital toxoplasmosis. Furthermore, our results suggested that the implementation of newborn screening and follow-up testing can lead to reducing of toxoplasmosis associated complications.

## Data Availability

The datasets generated and analyzed during the current study are not publicly available because they are archived in the database of the Hawassa University research and technology transfer Vice president Database Center and only are used for scientific purposes. Datasets may be available from the corresponding authors upon request.

## Abbreviations

ANC: Antenatal care
AOR: Adjusted odds ratio
EDHS: Ethiopian health and demographic survey
ELISA: Enzyme-linked immunosorbent assay
HIV: Human immunodeficiency virus
IgG: Immunoglobulin G
IgM: Immunoglobulin M
